# Oral Delivery of Lipophilic Drugs: The Tradeoff between Solubility Increase and Permeability Decrease When Using Cyclodextrin-Based Formulations

**DOI:** 10.1371/journal.pone.0068237

**Published:** 2013-07-16

**Authors:** Avital Beig, Riad Agbaria, Arik Dahan

**Affiliations:** Department of Clinical Pharmacology, School of Pharmacy, Faculty of Health Sciences, Ben-Gurion University of the Negev, Beer-Sheva, Israel; University of Quebect at Trois-Rivieres, Canada

## Abstract

The purpose of this study was to investigate the impact of oral cyclodextrin-based formulation on both the apparent solubility and intestinal permeability of lipophilic drugs. The apparent solubility of the lipophilic drug dexamethasone was measured in the presence of various HPβCD levels. The drug’s permeability was measured in the absence vs. presence of HPβCD in the rat intestinal perfusion model, and across Caco-2 cell monolayers. The role of the unstirred water layer (UWL) in dexamethasone’s absorption was studied, and a simplified mass-transport analysis was developed to describe the solubility-permeability interplay. The PAMPA permeability of dexamethasone was measured in the presence of various HPβCD levels, and the correlation with the theoretical predictions was evaluated. While the solubility of dexamethasone was greatly enhanced by the presence of HPβCD (K_1∶1_ = 2311 M^−1^), all experimental models showed that the drug’s permeability was significantly reduced following the cyclodextrin complexation. The UWL was found to have no impact on the absorption of dexamethasone. A mass transport analysis was employed to describe the solubility-permeability interplay. The model enabled excellent quantitative prediction of dexamethasone’s permeability as a function of the HPβCD level. This work demonstrates that when using cyclodextrins in solubility-enabling formulations, a tradeoff exists between solubility increase and permeability decrease that must not be overlooked. This tradeoff was found to be independent of the unstirred water layer. The transport model presented here can aid in striking the appropriate solubility-permeability balance in order to achieve optimal overall absorption.

## Introduction

Modern drug discovery techniques, including high-throughput *in vitro* screening methods of receptor binding and the introduction of combinatorial chemistry, are yielding an increasing number of lipophilic, pharmacologically active compounds [1], [2]. Due to their poor water solubility, the overall rate limiting factor in the oral absorption of these compounds is often their solubility/dissolution in the hydrophilic intestinal milieu. According to the Biopharmaceutical Classification System (BCS), drug candidates with low solubility and favorable permeability characteristics, would be classified as class II compounds [3], [4], [5], [6]. These compounds are frequently characterized by low oral bioavailability, and therefore fail to proceed to advanced stages of research and development [7], [8], [9].

A popular approach to improve the solubility of lipophilic drugs is the utilization of cyclodextrin-based formulations [10], [11]. Cyclodextrins are crystalline, nonhygroscopic, cyclic oligosaccharides, with a hydrophilic outer surface and a lipophilic central cavity. From a pharmaceutical standpoint, cyclodextrins have gained widespread attention and use due to their ability to interact with poorly water soluble drugs, resulting in an increase in their apparent water solubility.

It is generally accepted that neither free cyclodextrin nor drug-cyclodextrin complex are absorbed to an appreciable extent [11], [12]. Thus, following oral administration of cyclodextrin-drug complex, only the free drug is available for permeation and absorption. Association and dissociation of the free drug are often assumed to be rapid in comparison to the kinetics of dissolution and permeation [13], and hence, the effect of cyclodextrin complexation on the permeation of the drug through the GI wall due to reduced free drug concentration is often overlooked. However, this is not necessarily the case. Indeed, a critical review of the literature reveals that the use of cyclodextrins may lead to improved, unchanged, or even reduced overall absorption [14], [15]. This unpredicted effect may be attributable to the phenomenon that we have named the solubility-permeability interplay; several reports have emerged that while increasing the apparent solubility of the drug, cyclodextrins may on the same time reduce the apparent permeability of the co-administered drug [12], [16], [17], [18], [19], [20], [21], [22], [23].

In a recent series of publications, we have shown that a tradeoff may exist between solubility increase and permeability decrease when using solubility-enabling formulations, that can lead to paradoxical effects on the overall absorption [17], [22], [24], [25], [26], [27]. With respect to cyclodextrins, we have shown that decreased apparent intestinal permeability accompanied the increased solubility when using cyclodextrin-based formulation for the lipophilic drug progesterone. We have shown that the unstirred water layer (UWL) plays a significant role in progesterone’s absorption process, and developed a mathematical model that successfully simulated the effect of the cyclodextrin on the drug’s permeability through the UWL, the membrane, and the overall effective permeability [17].

**Figure 1:**
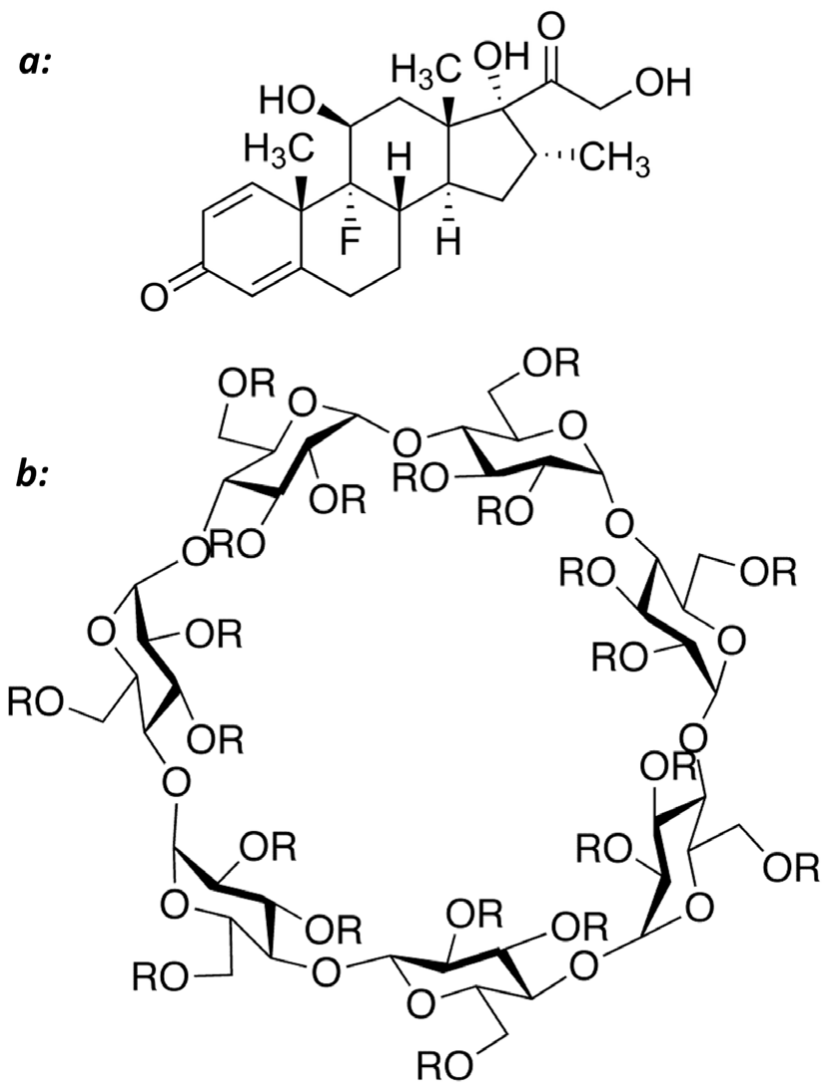
The molecular structures of dexamethasone (*a*) and 2-hydroxypropyl-β-cyclodextrin (*b*). R = -H or –CH_2_CHOHCH_3_.

The purpose of the present study was to investigate the effect of cyclodextrin complexation on the intestinal permeability of lipophilic drugs when the UWL does not play a significant role in the absorption process. The slightly water soluble high-permeability steroid dexamethasone (Figure 1) was selected as a model drug. The effect of 2-hydroxypropyl-β-cyclodextrin (HPβCD) on the drug’s solubility was studied, the role of the UWL on the absorption of dexamethasone was investigated in the Caco-2 model, and the intestinal permeability of dexamethasone in the absence vs. the presence of HPβCD was evaluated in various experimental methods: the parallel artificial membrane permeability assay (PAMPA), transport across Caco-2 cell monolayers, and the *in situ* single-pass intestinal perfusion model in rats. Overall, this setup allowed us to reveal the solubility-permeability interplay when using cyclodextrin-based formulations for oral administration of lipophilic drugs, and to enable a more efficient and intelligent use of molecular complexation strategies to facilitate successful oral drug delivery.

## Materials and Methods

### Materials

Dexamethasone, HPβCD, dexamethasone-HPβCD complex, phenol red, hexadecane, MES buffer (2- (N- morpholino) ethanesulfonic acid), HEPES buffer (4- (2- Hydroxyethyl) piperazine- 1- ethanesulfonic acid), KCl and NaCl were purchased from Sigma Chemical Co. (St. Louis, MO). n-Hexane was obtained from Frutarom LTD (Haifa, Israel). Acetonitrile, methanol and water (Merck KGaA, Darmstadt, Germany) were UPLC grade. All other chemicals were of analytical reagent grade.

### Solubility Studies

The solubility experiments for dexamethasone with HPβCD were conducted according to the method described by Higuchi and Connors [28]. Briefly, to a number of glass test tubes containing excess amounts of dexamethasone, 0–0.015 M HPβCD in MES buffer solutions (pH 6.5) were added. The test tubes were tightly closed and placed in a shaking water bath at 25°C and 100 rpm for 48 hours. Establishment of equilibrium was assured by comparison of samples after 24 and 48 hours. Before sampling, the vials were centrifuged at 12,000 rpm for 10 min. Supernatant was carefully withdrawn from each test tube, filtered, and immediately assayed for drug content by UPLC.

The dexamethasone-HPβCD binding constant (K_1∶1_) was calculated from the phase-solubility analysis using the following equation [28], [29]:
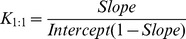
where the binding constant (K_1∶1_) is calculated from the slope-intercept form of the linear equation of the drug’s solubility as a function of increasing HPβCD concentration.

### Parallel Artificial Membrane Permeability Assay (PAMPA)

The PAMPA studies were carried out in 96-well MultiScreen Permeability Filter Plate obtained from Millipore Co. (Billerica, MA), using the hexadecane method described previously [30], with some modifications [31], [32]. Briefly, each well was impregnated with 15 µL of 5% hexadecane dissolved in n-hexane for 1 hr to allow complete evaporation of the hexane. Subsequently, the donor compartments were filled with 200 µL of 50 µg/mL dexamethasone in 0–0.01 M HPβCD MES buffer solution (pH 6.5), and connected to the acceptor plate which had been prefilled with 300 µL of similar blank buffer. The resulting sandwich construct was incubated at room temperature. Receiver plate wells were then collected every 30 min over 2.5 hours, and the dexamethasone concentration in each well was determined by UPLC. Five wells were loaded at each HPβCD level to enable collection at different time points (30–150 min) thereby tracking the permeability kinetics. Each experiment was repeated four times (*n = *4).

Apparent permeability coefficient (*P*_app_) was calculated from the linear plot of drug accumulated in the receiver side versus time, according to the equation:
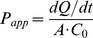
where d*Q*/d*t* is the steady-state appearance rate of the drug on the receiver side, *C*_0_ is the initial concentration of dexamethasone in the donor side, and A is the membrane surface area (0.048 cm^2^). Linear regression was carried out to obtain the steady-state appearance rate of dexamethasone on the receiver side.

### Cell Culture

Caco-2 cells (passage 27) from American Type Culture Collection (Rockville, MD) were routinely maintained in Dulbecco’s modified Eagle’s medium containing 10% fetal bovine serum, 1% nonessential amino acids, 1 mM sodium pyruvate, and 1% l-glutamine. Cells were grown in an atmosphere of 5% CO_2_ and 90% relative humidity at 37°C.

Caco-2 cells were seeded on semi-permeable filter inserts (6-well Transwell plate, Corning Costar Co., Cambridge, MA) at approximately 150,000 cells per filter (growth area 4.7 cm^2^). The cells on the insert were cultured for 21 days at 37°C in a humidified incubator containing 5% CO_2_ in air. The differentiation status of the formed monolayer was evaluated by measuring the transepithelial electrical resistance (TEER) (Millicell-ERS epithelial Voltohmmeter, Millipore Co., Bedford, MA). Following the 21 days in cell culture, the monolayers developed a TEER values above 250–300 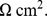


### Caco-2 Permeability Studies

Transcellular transport studies were performed as described previously [33], [34], [35] with minor modifications. Uptake buffers contained 1 mM CaCl_2_, 0.5 mM MgCl_2_·6 H_2_O, 145 mM NaCl, 3 mM KCl, 1 mM NaH_2_PO_4_, 5 mM d-glucose, and 5 mM MES. Briefly, 1.5×10^5^ cells were seeded onto collagen-coated membranes (0.4-µm pore size, 24 mm diameter, Costar, Cambridge, MA) and cells were allowed to grow for 21 days. Mannitol permeability was assayed for each batch of Caco-2 monolayers (*n = *3) and TEER measurements were performed on all monolayers. Monolayers with apparent [^14^C]mannitol permeability <3×10^−7^ cm/sec and TEER values >250 Ωcm^2^ were used for the study. Free dexamethasone or dexamethasone-HPβCD complex solution (1.5 mL) in MES buffer (pH 6.5) was added to the apical side of the monolayer and 2.6 mL of HEPES buffer (pH 7.4) to the receiver compartment on the basolateral side of the monolayer. The plates were then incubated at 37°C on 50 rpm orbital shaker. Samples were taken from both sides (200 µL from basolateral side, 20 µL from apical side) at various time points up to 120 min. All samples were immediately assayed for dexamethasone content. Apparent permeability coefficient (*P*_app_) was calculated as described above for the PAMPA studies. These experiments were repeated 4 times (*n* = 4).

The role of the unstirred water layer (UWL) in the absorption process of dexamethasone was investigated by assessing the effect of rotation speed on the drug’s Caco-2 transport [17]. The procedure described above for the Caco-2 permeability studies was repeated in 3 different rotation speeds: 0 (no shaking), 50, and 100 rpm. These experiments were repeated 3 times (*n* = 3).

### Single-Pass Rat Intestinal Perfusion Studies

All animal experiments were conducted using protocols approved by the Ben-Gurion University of the Negev Animal Use and Care Committee (Protocol IL-60-11-2010). Animals were housed and handled according to Ben-Gurion University of the Negev Unit for Laboratory Animal Medicine Guidelines. Male Wistar rats (Harlan, Israel) weighing 250–300 g were used for all studies. Prior to each experiment, the rats were fasted overnight (12 h) with free access to water. Animals were randomly assigned to the different experimental groups.

The procedure for the *in situ* single pass perfusion followed previously published reports [36], [37], [38]. Briefly, rats were anesthetized with an intra-muscular injection of 1 mL/kg of ketamine-xylazine solution (9%:1%, respectively) and placed on a heated surface maintained at 37°C (Harvard Apparatus Inc., Holliston, MA). The abdomen was opened by a midline incision of 3 cm. A jejunal segment of approximately 10 cm was carefully exposed and cannulated on two ends with flexible PVC tubing. The dexamethasone or dexamethasone-HPβCD complex perfusion solution (10 mM MES buffer, pH 6.5, 135 mM NaCl, 5 mM KCl, and 0.1 mg/mL phenol red, with an osmolarity of 290 mosm/L) was incubated in a 37°C water bath, and was pumped through the cannulated intestinal segment at a flow rate of 0.2 ml/min (Watson-Marlow 205S, Wilmington, MA). The perfusion buffer was first perfused for 1 hour, in order to ensure steady state conditions. After reaching steady state, samples were taken in 10 min intervals for one hour. All samples were immediately assayed for drug content by UPLC. Following the termination of the experiment, the length of each perfused jejunal segment was accurately measured.

The effective permeability (*P_eff_*) through the rat gut wall in the single-pass intestinal perfusion studies was determined using the following equation:
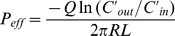
where *Q* is the perfusion buffer flow rate, *C′_out_*/*C′_in_* is the ratio of the outlet and the inlet concentration of dexamethasone that has been adjusted for water transport, *R* is the radius of the intestinal segment (set to 0.2 cm), and *L* is the length of the perfused intestinal segment.

### Ultra Performance Liquid Chromatography (UPLC)

UPLC analyses were performed on a Waters (Milford, MA) Acquity UPLC H-Class system equipped with photodiode array detector and Empower software. Dexamethasone was assayed using a Waters (Milford, MA) Acquity UPLC BEH C_18_ 1.7 µm 2.1 × 100 mm column. The detection wavelength was 240 nm. The mobile phase consisted of 30∶70 going to 70∶30 (v/v) 0.1% TFA in water: 0.1% TFA in acetonitrile over 5 minutes, and was pumped at a flow rate of 0.5 mL/min. Injection volumes for all UPLC analyses ranged from 5 to 50 µL.

### Statistical Analysis

All *in vitro* experiments were conducted three to four times, and all animal experiments were performed four times (*n* = 4). Values are expressed as means ± standard deviation (SD). To determine statistically significant differences among the experimental groups, the nonparametric Kruskal-Wallis test was used for multiple comparisons, and the two-tailed nonparametric Mann–Whitney U-test for two-group comparison where appropriate. A *p* value of less than 0.05 was termed significant.

## Results

### Phase-Solubility Studies

**Figure 2:**
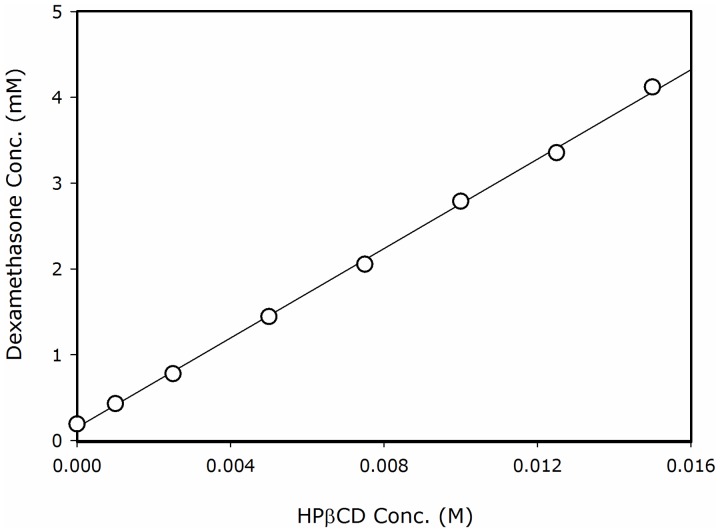
Aqueous solubility of dexamethasone as a function of increasing HPβCD concentration at 25°C. Data are presented as mean ± SD (error bars smaller than symbols); *n* = 3 in each experimental group.

Phase-solubility diagram of dexamethasone with HPβCD in 25°C is shown in Figure 2. The solubility diagram was found to be of Higuchi A_L_-type, i.e. linear increase was observed with unchanged stoichiometry [28]. The linear A_L_-type diagram suggests that the complexation of dexamethasone and HPβCD is predominantly a 1∶1 complex within the range of concentrations studied. A binding constant (K_1∶1_) of 2311 M^−1^ was calculated from the phase-solubility diagram.

### Caco-2 Permeability Studies

**Figure 3:**
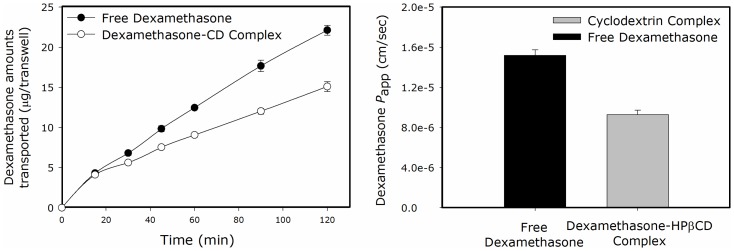
Dexamethasone flux across Caco-2 monolayers as the free drug (•) vs the drug-HPβCD complex (○), and the corresponding P_app_ values in centimeters per second (right panel). Data are presented as mean ± SD; *n* = 4 in each experimental group.

Apical to basolateral permeability was investigated for dexamethasone as the free drug vs. dexamethasone-cyclodextrin complex. The drug’s flux and apparent permeability coefficients across Caco-2 cell monolayers obtained for the two cases are presented in Figure 3. The data demonstrate that the permeation of the drug across Caco-2 monolayers was significantly decreased following complexation with cyclodextrin in comparison to the free drug solution, with doubled permeability coefficient (*P*_app_) value.

**Figure 4:**
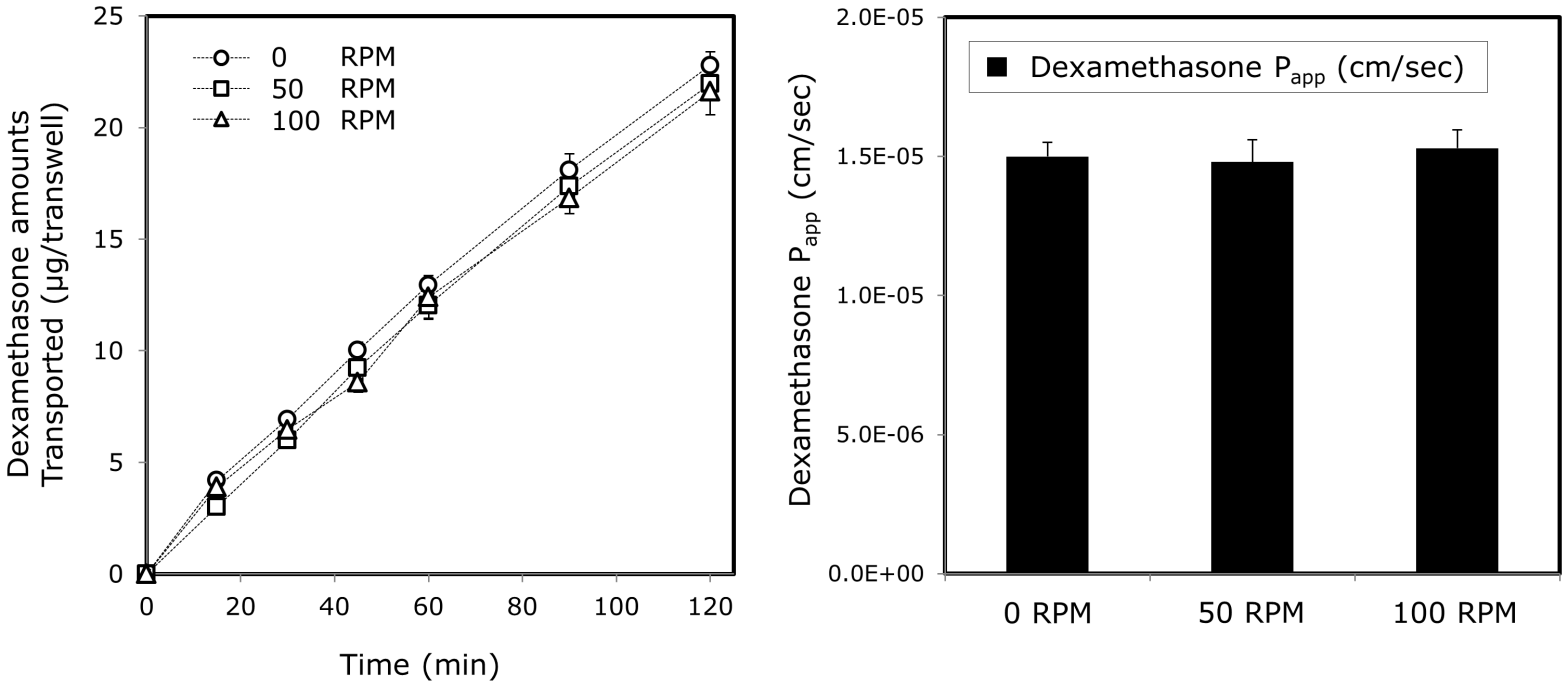
P_app_ values of dexamethasone across Caco-2 monolayers at different rotation speeds (0, 50, and 100 rpm). Data are presented as mean ± SD; *n* = 4 in each experimental group.

The role of the unstirred water layer (UWL) in the absorption process of dexamethasone was investigated by assessing the effect of rotation speed on the drug’s Caco-2 transport [17]. The dependence of dexamethasone’s Caco-2 permeability on the rotation speed is presented in Figure 4. It can be seen that, unlike our previous report with progesterone [17], dexamethasone showed similar flux and permeability coefficients across Caco-2 for all three rotation speeds studied, indicating the lack of UWL effect in the intestinal absorption process of dexamethasone.

### Rat Perfusion Studies

**Figure 5:**
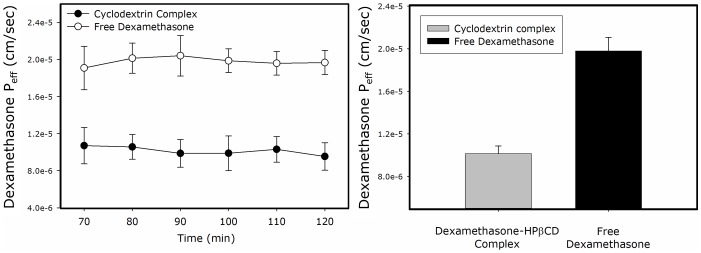
Effective permeability values (P_eff_, cm/sec; right panel) and outlet/inlet concentration ratio (C′_out_/C′_in_; left panel) of dexamethasone as the free drug (○) vs the drug-HPβCD complex (•), determined using the *in situ* single-pass rat jejunal perfusion model. Data are presented as mean ± SD; *n* = 4 in each experimental group.

The effective permeability coefficients obtained for dexamethasone as the free drug vs. dexamethasone-HPβCD complex in the *in situ* single-pass rat intestinal perfusion model are presented in Figure 5. Again, similarly to the Caco-2 results, a twofold lower intestinal permeability was evident for dexamethasone in the presence vs. the absence of HPβCD.

### Predicted vs. Experimental PAMPA Permeability

The lack of UWL effect in the absorption process of dexamethasone revealed by the Caco-2 studies essentially indicates that the intestinal transport of dexamethasone is effectively membrane controlled. This finding greatly simplifies the transport model that describes and predicts the solubility-permeability interplay; since no effective aqueous boundary layer is involved, the intrinsic effective permeability, that is, in the absence of HPβCD, represents the true membrane permeability of the drug, and as we have shown before [17], the dependence of dexamethasone permeability on HPβCD level may be predicted directly from the proportional increase in the apparent solubility, according to the equation:
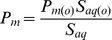
where *P*_m_, the apparent membrane permeability at a given HPβCD level equal to the intrinsic membrane permeability (*P*_m(o)_) corrected to the ratio between the intrinsic solubility i.e. in the absence of HPβCD (*S*_aq(o)_) to the apparent solubility at a given HPβCD level (*S*_aq_).

**Figure 6:**
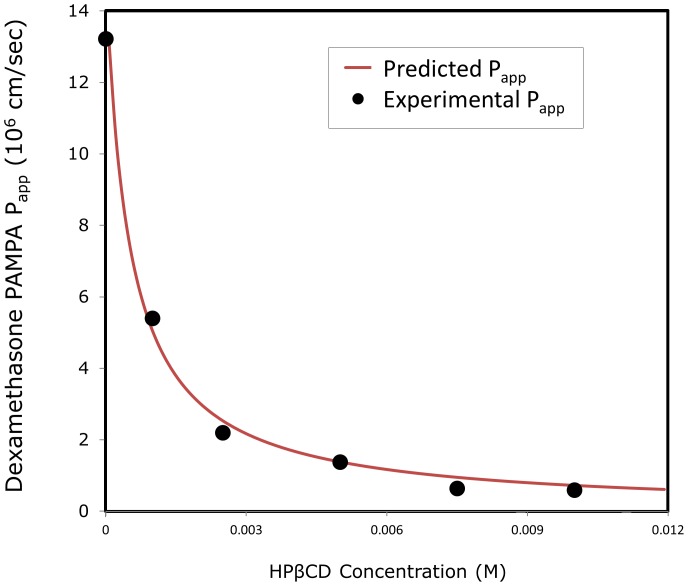
Theoretical vs. experimental apparent permeability (P_app_; cm/sec) of dexamethasone in the PAMPA model as a function of increasing HPβCD levels. Experimental data presented as mean ± SD (error bars smaller than symbols); *n* = 4.

Comparison of the predicted *P*_app_ of dexamethasone as a function of HPβCD level according to the equation above to the experimentally obtained *P*_app_ values is presented in Figure 6. The experimental values used in the calculations were the solubility in the absence vs. presence of different HPβCD concentrations (Figure 2). The permeability in the absence of HPβCD was taken as the value for dexamethasone’s true membrane permeability, as explained above. It can be seen that excellent agreement was obtained between the experimental and the predicted *P*_app_ values of dexamethasone as a function of HPβCD level (Figure 6).

## Discussion

**Figure 7:**
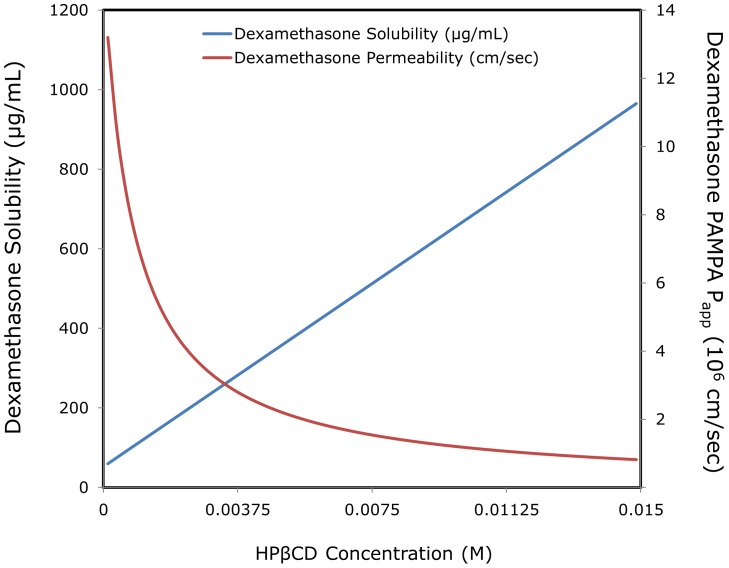
The overall effects of increasing HPβCD levels on dexamethasone apparent solubility and permeability, based on the mass transport model employed in this work.

The focus of this work is the tradeoff between the solubility increase and permeability decrease when employing cyclodextrin-based formulations to improve the oral absorption of lipophilic drugs. We have shown this tradeoff before in a situation where the UWL played a significant role in the absorption process of the drug [17], [22], [25]; here, we show that the solubility-permeability tradeoff occurs irrespectively of the UWL. Moreover, the lack of UWL effect greatly simplifies the description of the solubility-permeability interplay, allowing to predict the decreased apparent permeability directly from the proportional increase in the apparent solubility. The overall effect of HPβCD on the apparent solubility and permeability of dexamethasone is illustrated in Figure 7, according to the simplified mass transport model applied in this work. This figure visibly illustrates the opposing effects that cyclodextrins may have on the apparent solubility and intestinal permeability. Given these opposing effects, both apparent solubility and permeability considerations must be taken into account, to strike the appropriate balance in order to achieve optimal absorption from a cyclodextrin-based formulation.

The mechanism behind the decreased permeability that accompanies the increased solubility when using cyclodextrin-based formulation may be attributable to the way by which cyclodextrins allow to increase the solubility of lipophilic drugs. By hosting the drug in the hydrophobic central core and forming a complex with higher aqueous solubility in comparison to the free drug, the cyclodextrin-based formulation also decreases the free fraction of the drug. Decreased free fraction is directly translated to lower concentration gradient and hence thermodynamic driving force for membrane permeation. Therefore, the decreased permeability with increased apparent solubility could be attributed to free fraction considerations. This rationale is also applicable to surfactant-based formulations [27]. However, we have then shown that the solubility-permeability tradeoff is also obtained when using cosolvent-based formulations, in which free-fraction considerations are not relevant [24], [26]. Therefore, we have shown that the root of the solubility-permeability tradeoff is deeper than free-fraction considerations, and in fact goes back to the definition of permeability. Intestinal permeability is equal to the diffusion coefficient of the drug through the membrane times the membrane/aqueous partition coefficient of the drug divided by the membrane thickness. Hence, increasing the apparent solubility of the drug in the aqueous medium *via* formulation will decrease the membrane/aqueous partition coefficient of the drug, directly leading to decreased apparent intestinal permeability. These opposing effects on apparent solubility and permeability must be taken into account in order to fully understand the impact on the overall fraction of drug absorbed when solubility-enabling formulations are employed to enhance the oral exposure of a poorly soluble drug. We have recently revealed that the solubility-permeability tradeoff can be circumvented by using amorphous solid dispersions that increase the apparent solubility of the drug by supersaturation (and not solubilization), provided substantial supersaturation can be achieved and maintained [39], [40]. However, the development and manufacture of these solubility enabling formulations are much more challenging.

### Conclusions

This work demonstrates that when using cyclodextrins as pharmaceutical solubilizers, a tradeoff exists between solubility increase and permeability decrease that must not be overlooked. This tradeoff was found to be independent of the unstirred water layer. The simplified transport model presented here can aid in striking the appropriate solubility-permeability balance in order to achieve optimal overall absorption.
