# Correction: Oral Delivery of Lipophilic Drugs: The Tradeoff between Solubility Increase and Permeability Decrease When Using Cyclodextrin-Based Formulations

**DOI:** 10.1371/annotation/10ae05dc-90cb-46f1-aa2b-e63eb4a132e2

**Published:** 2013-10-23

**Authors:** Avital Beig, Riad Agbaria, Arik Dahan

There was an error in the Competing Interests statement. The Competing Interests statement should read: "The work was supported by a research grant from AbbVie Incorporation. This does not alter the authors' adherence to all the PLOS ONE policies on sharing data and materials." 

